# Effects of climate and potential policy changes on heating degree days in current heating areas of China

**DOI:** 10.1038/s41598-018-28411-z

**Published:** 2018-07-05

**Authors:** Ying Shi, Guiling Wang, Xuejie Gao, Ying Xu

**Affiliations:** 1 National Climate Center, China Meteorological Administration, Zhongguancun Nandajie 46, Haidian District, Beijing, 100081 China; 2 Department of Civil and Environmental Engineering, and Center for Environmental Sciences and Engineering, University of Connecticut, Storrs, CT 06269 USA; 3 Climate Change Research Center, Institute of Atmospheric Sciences, Chinese Academy of Sciences, Huayanli 40, Chaoyang District, Beijing, 100029 China

## Abstract

Based on climate simulations over East Asia from a high-resolution regional climate model under RCP4.5 and 8.5 scenarios, we examine the impact of future climate change and heating policy changes on energy demand in current central heating areas over China using the heating degree days (HDD) and the number of the heating days (NHD) with different base temperature as the indices. Based on current heating policy in China, significant decreases of NHDs are projected, with larger decreases under RCP8.5 than RCP4.5. This decrease of NHDs would cause a northward shift of the decadal heating boundary line, with significant implications for infrastructure planning and development. Changing the heating policy currently in practice to one used in Europe and USA would cause an immediate jump in NHDs and in HDDs; as warming progresses in the future, these effects attenuate with time in an approximately linear trend under the two scenarios. Under RCP8.5, by 2050, the effects of warming climate would dominate over the heating policy change, and heating demand would be lower than the present day HDD and continue to decrease until the end of the century. Energy demand and the number of the heating days during peak winter shows no dependence on heating policy, as the policy-induced increase of energy demand would occur primarily during warmer months of the year. In addition, the indices are further weighted by population, and results show that increases in both HDDs and NHDs can be found in parts of northern China due to the increased population there by the end of the 21st century.

## Introduction

Warming of the climate system is unequivocal. Global average of surface temperature shows a warming of 0.85 [0.65 to 1.06] °C over the period of 1880 to 2012^1^. Over China, the observed warming is in the range of 0.9 to 1.5 °C from 1909, with a rate larger than the global mean^2^. Continued emission of greenhouse gases (GHGs) will cause further warming, which is estimated to range from 0.3 to 4.8 °C in global average by the end of the century (2081–2100) relative to the present day (1986–2005)^1^. In China, this range is approximately 1.4 to 5.1 °C^3^. In the context of global warming, changes in climate have influenced, and will continue to influence natural and human systems^4^, which in many cases induce feedback to carbon cycles with significant implications. For example, energy consumption for heating and cooling in the household sector is directly related to temperature, accounting for nearly 20% of the total energy consumption (therefore a major fraction of CO_2_ emission) in China^5^. Of that, up to 85% is the heating in the north central heating areas and is mainly produced from coal burning, which can lead to serious problems of air pollution^6^. Understanding how this section of China’s energy demand (especially for the heating energy consumption) might change in the future is therefore important and valuable for energy supply planning and management as well as pollution control.

Warmer climate can lead to a decrease in heating demand and an increase in cooling demand^7,8^. The simplest way to estimate the household energy consumption is through the concept of degree day which is developed by Thom^9,10^ and based on daily mean temperature. As the parameter is easy to calculate, it has been widely used in climate change studies at both global and regional scales^11–17^. The definition of both heating and cooling degree days (HDD and CDD, respectively) involves a temperature threshold, and the threshold used in specific applications varies according to human physiological needs, energy supply, economic level, temperature characteristics and so on. For example, the threshold temperature for HDD and CDD employed are 13 °C and 23 °C in Spain^18^, 18 °C and 22 °C in Europe^19^, and both are 18.33 °C for the United States^20^. The reference temperature used for China also differs among different studies^16,17,21,22^, but 5 °C has been widely used for defining the starting and ending dates of HDD for historical policy reasons (the heating starts (ends) when temperature is below (above) 5 °C for a continuous 5-day period, see next paragraph and Methods for more detail) and 18 °C for calculating the HDD in the heating period. Shi *et al*.^17^ showed that by the mid-century, the population-weighted HDD under RCP4.5 scenario would be 261 °C·d lower than the present-day if the current practice continues, and would be 749 °C·d higher than present-day if the developed world standard of 18 °C was used as the threshold temperature.

To accurately reflect energy demand, the calculation of degree days has to account for the common practice in the specific region or country of interest. China’s heating system is different from what is used elsewhere in the world. Because of historical energy shortage, central heating is only available in northern China in cold season, and the generally accepted boundary line is the Qinling-Huaihe line (around 32~34°N in the eastern part of China). Only areas north of this line are equipped with central heating facilities^23^ and people are not allowed to individually control heating in their own space. South of this line, no central heating is provided, and people start to use air conditioners or individual space heaters during winter following the economic development in recent decades. Although several extreme snow and ice storms in southern China during the past ten years have triggered discussions on the potential to install central heating in the south, the cost related to heating facilities, pipeline networks, and building upgrading is prohibiting^24^. In South China, the heating energy demand can be up to 8~12 kilograms (KG) of coal equivalent for each winter when using the central heating system while that is only about 5 KG of coal equivalent when using individual space heaters. For this reason, Jiang^24^ suggested that individual heating is more suitable for it.

To guide future energy management and planning and to inform climate adaptation and mitigation strategies development, this study assesses the impact of future climate and potential heating policy changes on energy consumption in current central heating areas based on a high-resolution regional climate simulation under RCP4.5 and 8.5 scenarios over East Asia. The simulations are conducted using a recent version of Abdus Salam International Center for Theoretical Physics (ICTP) Regional Climate Model, RegCM4^25^, driven by boundary conditions from the Beijing Climate Center Climate System Model v.1.1 (BCC_CSM1.1)^26,27^. The simulation covers the period of 1951–2005 for the present day climate (with observed GHG concentrations) and 2006–2099 for the future under the RCP4.5 and RCP8.5 scenarios^28^, respectively. Extensive validations of the model performance based on several variables (temperature, precipitation, and extremes) were already conducted by others in earlier studies^17,29,30^ and results show that the model can reproduce the present day climate over the region reasonally well, significantly better than the driving GCM. For example, temperature bias is mostly within ± 1.0 °C in eastern China in RegCM and is generally in the range of 1.0 to 2.5 °C in the GCM. In this study we use energy consumption indices including the heating degree days (HDD) and the number of the heating days (NHD), and these indices are defined for both the current heating practice in China (HDD5 and NHD5; see Method for definition) and the base temperature 18 °C used in developed countries (HDD18 and NHD18; see Method for definition). In addition, changes of the starting and ending dates of the heating period (DSH and DEH) as well as the boundary line for heating areas are also assessed. The focus of analysis is over the present concentrated heating areas of China, including most of the provinces in northeastern, northern, northwestern and western China, and the projected future changes are defined based on the present-day period of 1986–2005 and the end of the 21st century 2080–2099.

## Results

### Validation of the climate model

**Figure 1:**
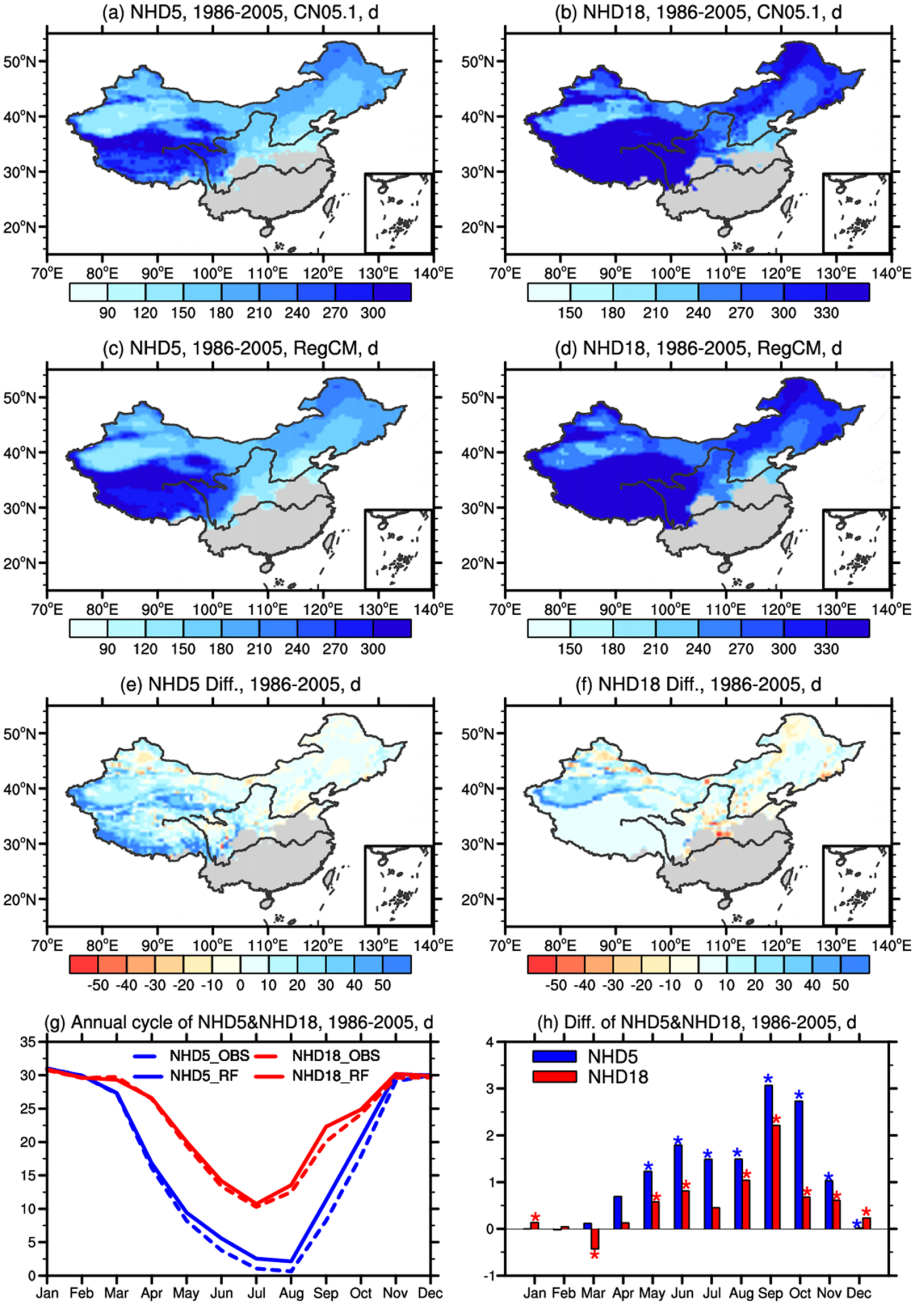
Annual mean observed and simulated (**a**,**c**) NHD5 and (**b**,**d**) NHD18, the model bias (model minus observation) (**e**,**f**), and the annual cycle (**g**) and bias (**h**) of regional mean NHD5 and NHD18 over the heating areas in the present day (1986–2005) (unit: d). Gray indicates no heating in the present day. The histograms highlighted with a blue (red) star for NHD5 (NHD18) are statistically significant at 95% confidence level. The maps were plotted with NCL 6.2.0 (free software; http://www.ncl.ucar.edu/).

Figure 1a–f shows the NHD5 and NHD18 over China for the period of 1986 to 2005 based on observed and RegCM4-simulated climates, along with the difference between the model simulation and observations (or model bias). Note that the observational dataset used in this study is CN05.1 with 0.5° × 0.5° (longitude-latitude) resolution, which is developed by Wu and Gao^31^. Results show that RegCM4 simulates reasonable number of NHD5 and NHD18, with the spatial correlation coefficients between simulated and observed data of 0.96 and 0.97, respectively (both statistically significant at the 95% confidence level). Significant biases can be found in specific regions. Compared with observational data, the model generally overestimates the number of the heating days based on NHD5, with a maxima bias of up to 50d over the Tibetan Plateau because of the model cold biases there. Some underestimation can be found in the northeastern and northwestern China due to the warm biases of the model in those regions^29^. For NHD18, the model mainly overestimates it in the western part and underestimates it in the eastern part. The maxima bias exceeding 50d can be found over northwestern China. Other regions see a much closer comparison between model and observations, with biases in the range of ±10d. The root mean square error (RMSE) for NHD5 and NHD18 are 26d and 16d, respectively.

Annual cycles of NHD5 and NHD18 averaged over the heating areas in China and the model bias are presented in Fig. 1g,h. As China is mainly located in the mid and high latitudes, the concentrated heating period is concentrated in the boreal winter (October to March) even though the starting and ending dates are different among provinces. Both the observed and simulated values of NHD5 and NHD18 are greater than 25d in most of the winter months (from November to March), with a maximum value of 31d for both NHD5 and NHD18 in January. During the rest of the year, NHD18 is greater than NHD5 because some of the days colder than 18 °C occur before the heating start date and/or after the heating end date and are therefore excluded from the NHD5 calculation. The difference between simulation and observation are in the range of −1d to 4d. The maxima of 3.1d and 2.2d, respectively, can be found in September in NHD5 and NHD18, which is a warm-to-cold transition month. Note that the value of NHD5 and NHD18 in NDJF (November-December-January-February) are almost the same, as these are the coldest months of a year and daily temperature is well below the reference temperature (so all days are included).

**Figure 2:**
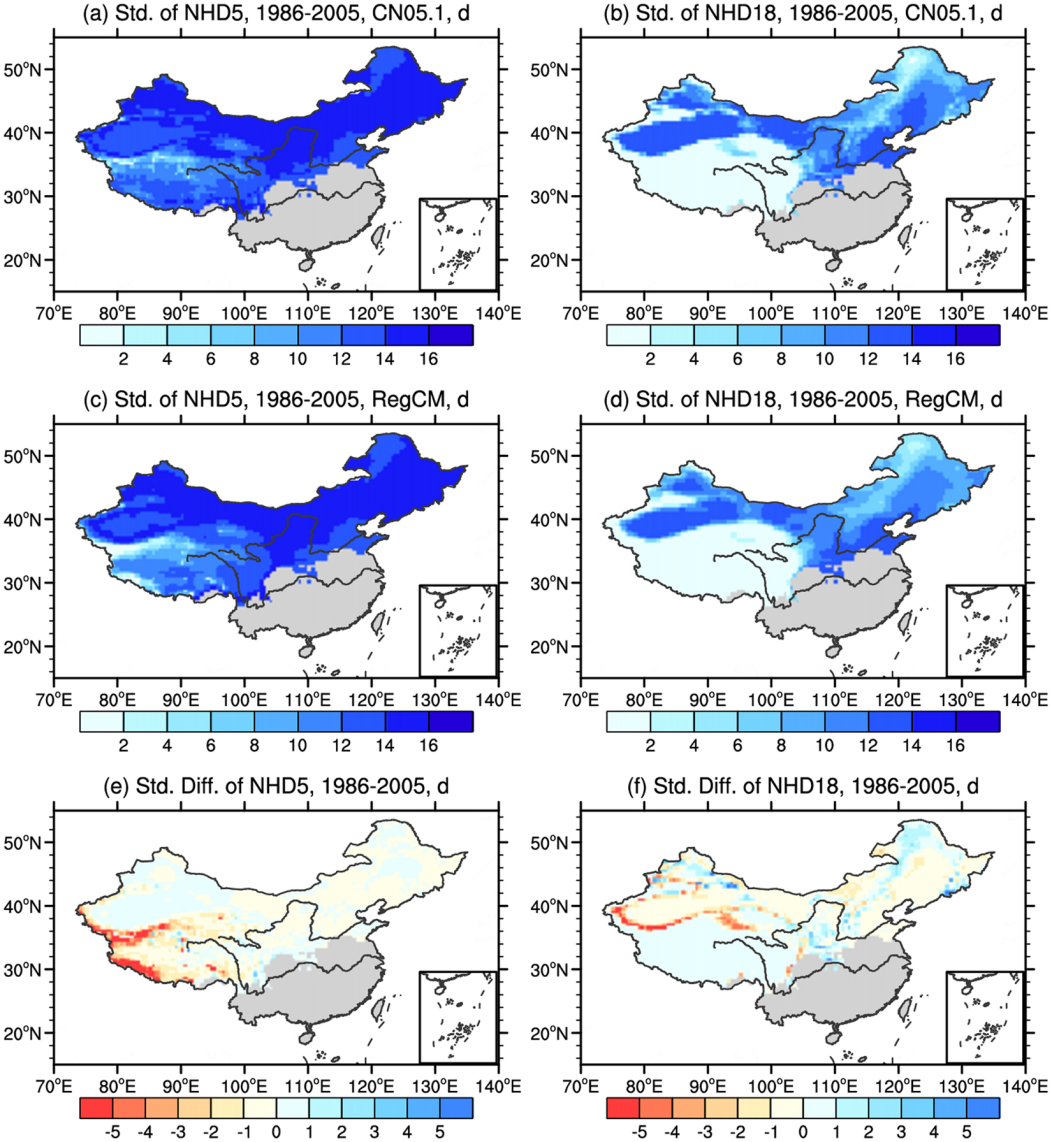
Interannual variability as measured by the interannual standard deviation for the present day (1986–2005). Observed and simulated (**a**,**c**) NHD5 and (**b**,**d**) NHD18, and the difference between simulation and observation (**e**,**f**) (unit: d). Gray indicates no heating in the present day. The maps were plotted with NCL 6.2.0 (free software; http://www.ncl.ucar.edu/).

Figure 2 shows the observed and simulated present-day interannual variability of NHD5 and NHD18, as measured by the interannual standard deviation. The model can reproduce the observed patterns of interannual variability well, with the spatial correlation coefficients of 0.81 and 0.96, respectively. For NHD5, significant differences can be found over the Tibetan Plateau, especially for the mountainous areas (the Himalaya and Kunlun Mountain). However, in NHD18, the simulated magnitudes are generally in line with the observation, with high values in the relative warm areas.

### Future changes of NHD5 and NHD18

**Figure 3:**
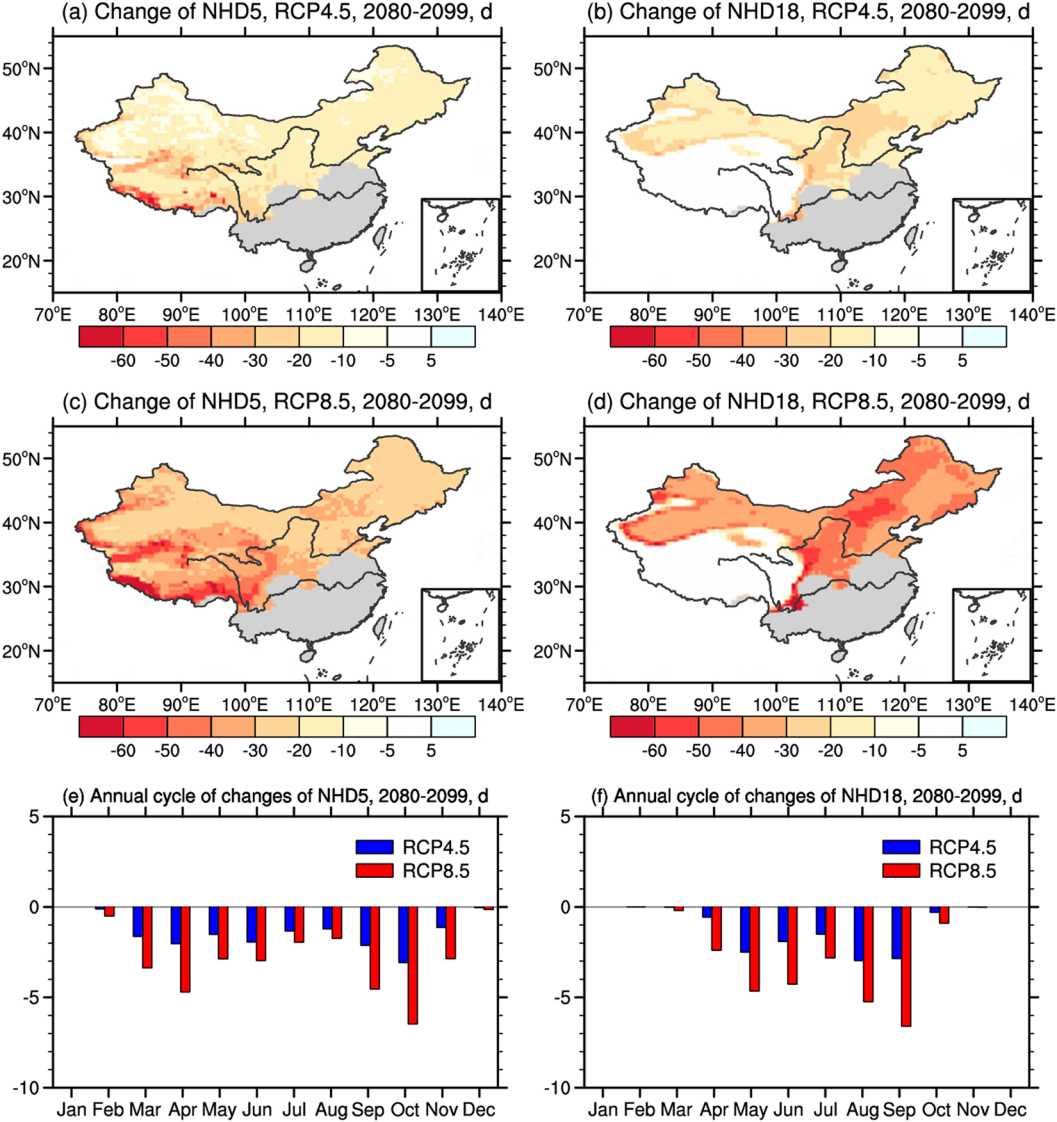
Changes of (**a**,**c**) NHD5 and (**b**,**d**) NHD18, and annual cycle of changes of (**e**) NHD5 and (**f**) NHD18 over the heating areas in the end (2080–2099) of the 21st century under RCP4.5 and RCP8.5 compared to the present day (1986–2005) (unit: d). The maps were generated using NCL 6.2.0 (free software; http://www.ncl.ucar.edu/).

Changes of NHD5 and NHD18 at the end of the 21st century relative to the present day (Fig. 3) can be attributed to climate change. A predominant decrease can be found in both NHD5 and NHD18, with greater change under RCP8.5. More specifically, the decrease of NHD5 is larger in the high-elevation areas with cold climate conditions and large expected warming^29^. Under RCP8.5, the decrease exceeds 30d in most of the concentrated heating areas, while under RCP4.5, a decrease of 20d or more can only be found over the Tibetan Plateau (Fig. 3a,c). The spatial distribution of NHD18 shows slight differences from NHD5, with the maximum decrease exceeding 30d and 50d under RCP4.5 and RCP8.5, respectively, over North China and the areas adjacent to the Tibetan Plateau (Fig. 3b,d). This is in line with You *et al*. (2014), in which the number of heating days is defined as the number of days when the daily mean temperature is lower than 24 °C. Regional mean changes of NHD5 and NHD18 over the heating areas for RCP4.5 are −16d and −12d, while those for RCP8.5 are −32d and −27d, respectively.

The annual cycle of the changes in NHD5 and NHD18 over the heating areas by the end of the 21st century are shown in Fig. 3e,f. Decrease of NHD5 and NHD18 are found in most of the months, with greater value of the decrease under RCP8.5. The maxima decrease (−6.5d) of NHD5 can be found in October, and the maximum decrease of NHD18 is expected in September (−6.6d), both of which are warm-to-cold transition months. The decrease in summer months (June, July and August) are relatively small, mainly in the range of 1~5d in both NHD5 and NHD18. Changes in winter months (December, January and February) are close to 0, as the daily temperature are well below the reference temperature even with future warming.

**Figure 4:**
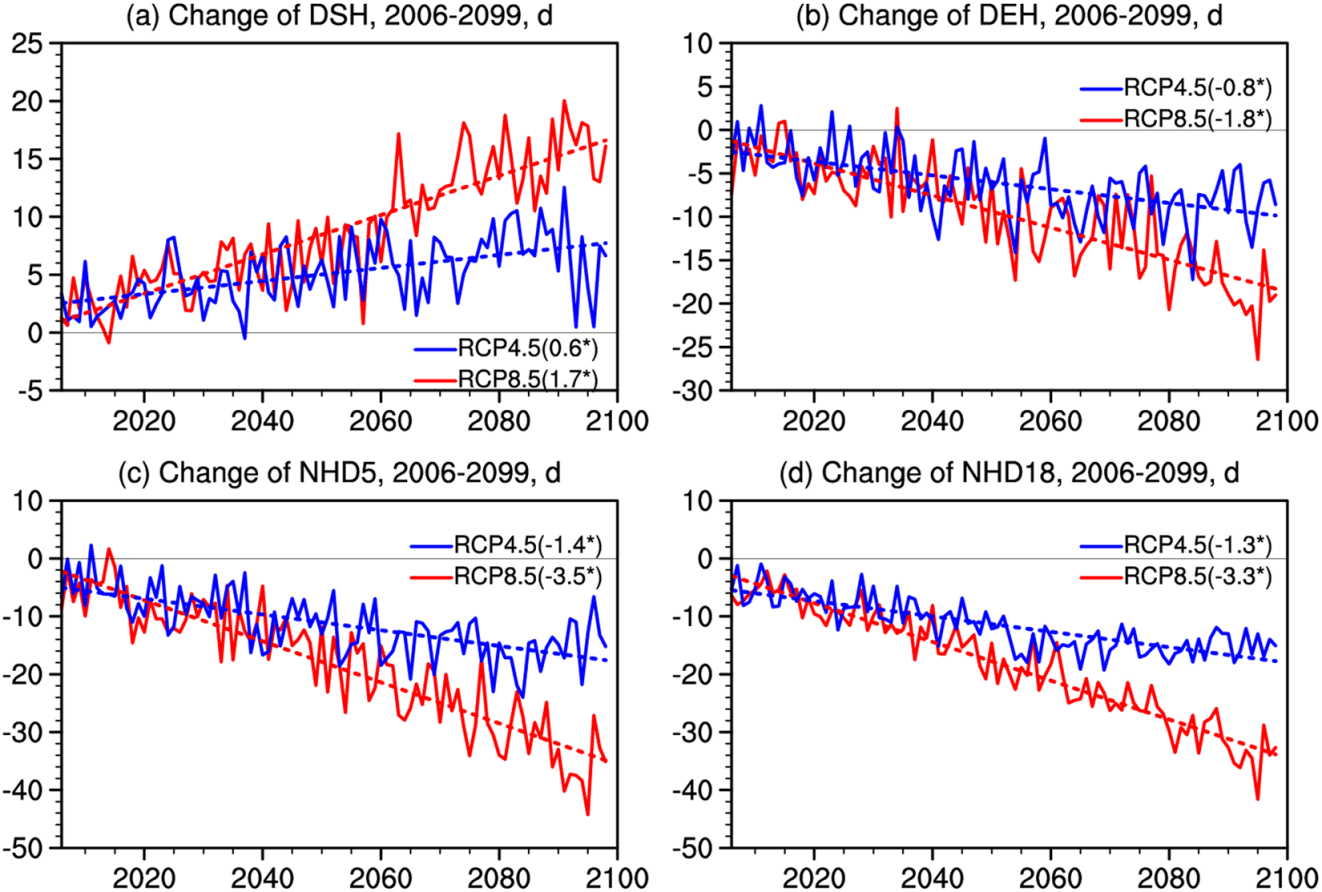
Changes of (**a**) DSH, (**b**) DEH, (**c**) NHD5 and (**d**) NHD18 averaged over the heating areas of China (2006–2099) under RCP4.5 and RCP8.5 compared to the present day (1986–2005) (unit: d). The trends of the changes are presented in parentheses (unit: d/10a; all significance at the 95% confidence level).

The temporal evolution of changes in the mean starting and ending dates of HDD5 (DSH and DEH), the heating durations (NHD5 and NHD18) under the RCP4.5 and RCP8.5 scenarios are presented in Fig. 4a–d. In general, changes of DSH, DEH, NHD5 and NHD18 before 2040 show little differences between RCP4.5 and RCP8.5, in agreement with the low scenario dependence of climate change in the early 21st century^1^; the projected change are more pronounced under RCP8.5 in the latter half of the century. Specifically, changes in DSH show a stepwise delay in the future and the linear trends are 0.6 and 1.7 d/10a under RCP4.5 and RCP8.5, respectively (Fig. 4a). Advanced DEH are found (Fig. 4b), corresponding to the reduction of the heating period (NHD5, Fig. 4c). The trends are −0.8 and −1.8 d/10a for DEH, for the two scenarios respectively, and −1.4 and −3.5 d/10a for NHD5. Significant decreases under the two scenarios can also be found in NHD18 (Fig. 4d), with linear trends of −1.3 and −3.3 d/10a for RCP4.5 and RCP8.5, respectively. Note that the linear trends for NHD18 are almost the same as that of NHD5, which is mainly caused by the similar value of NHD5 and NHD18 changes in the months from November to March (Fig. 3e,f). All trends shown in Fig. 4 are statistically significant at the 95% confidence level.

### Changes reflected by NHD18_5 and HDD18_5

**Figure 5:**
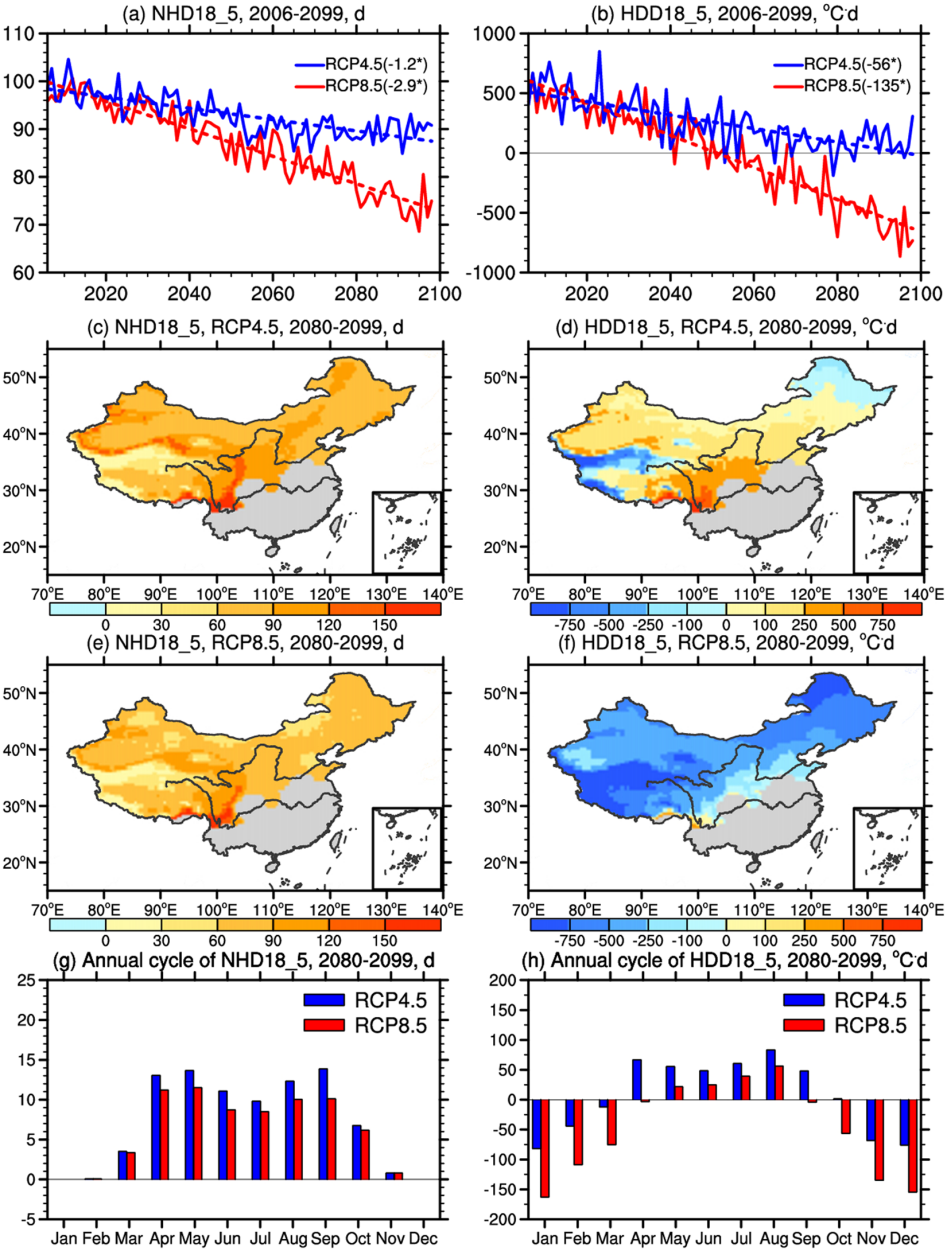
Changes of (**a**) NHD18_5 and (**b**) HDD18_5 during the period of 2006–2099, as well as (**c**,**e**) NHD18_5, (**d**,**f**) HDD18_5, and annual cycle of changes of (**g**) NHD18_5 and (**h**) HDD18_5 in the end (2080–2099) of the 21st century over the heating areas under RCP4.5 and RCP8.5 compared to the present day (1986–2005) (unit: d&°C·d). The trends of the changes in (**a**,**b**) are presented in parentheses (unit: d/10a&°C·d/10a; all significance at the 95% confidence level). The maps were generated using NCL 6.2.0 (free software; http://www.ncl.ucar.edu/).

The afore discussed future changes of NHD5 and NHD18 are caused by climate change; in the meanwhile, the national policy for heating might also change as the economy further develops. In this study, the future NHD18 (HDD18) is used to represent the probable future practice and the present NHD5 (HDD5) is used to represent the present-day practice. Therefore, the differences between the simulated NHD18 (HDD18) for the future (2080–2099) under RCP4.5/RCP8.5 and the simulated NHD5 (HDD5) for the present-day climate (1986–2005), hereafter referred to as NHD18_5 (HDD18_5), can be regarded as the changes caused by the combination of climate and perspective policy changes (Fig. 5). Similar to that in NHD5 and NHD18, a linear decreasing trend in NHD18_5 during the period of 2006–2099 is also presented with greater value under RCP8.5 (Fig. 5a), but the values are all positive under both scenarios which indicates the number of the heating days will increase in the future due to the policy changes. A linear decreasing trend is also observed in HDD18_5 (Fig. 5b), and most of the values are positive before 2050 s under both scenarios, suggesting more energy demand in the future (due to the dominant effects of policy changes). After 2050 s, the change of heating energy demand would stay at a relative stable level (around 150 °C·d) under RCP4.5, but would go from positive to negative under RCP8.5 (reaching −732 °C·d by the end of the century), signifying the effect of climate change dominant over the effect of policy change.

For the spatial distribution, under RCP4.5 and RCP8.5 by the end of the century, NHD18_5 is projected to increase in all concentrated heating areas, with a smaller magnitude under RCP8.5 (Fig. 5c,e). HDD18_5 under RCP4.5 suggests that the heating energy demand would decrease in the northern part of Northeast China and west of the Tibetan Plateau, which results from the greater warming that dominates over the impact of policy changes (Fig. 5d). Under RCP8.5, the decrease would further extend to most heating areas except for regions along the heating boundary (Fig. 5f). The greatest decrease can be found in the northern part of Northeast China and west of the Tibetan Plateau due to the greater warming there compared to other regions (figures not shown). Regional mean changes of NHD18_5 and HDD18_5 over the heating areas for RCP4.5 are 85d and 98 °C·d, while that for RCP8.5 are 71d and −533 °C·d, respectively.

Monthly regional mean changes reflected by NHD18_5 over the heating areas in China and the corresponding HDD18_5 by the end of the 21st century are shown in Fig. 5g,h, respectively. NHD18_5 indicates an increase of heating days in most of the months, with greater value of the increase under RCP4.5. It shows less change (close to 0) during November to February, similar to that of NHD5 and NHD18, as the daily temperature are well below the reference temperature even when under future warming. The maxima increase of NHD18_5 is 13.9d in September and 11.5d in May under RCP4.5 and RCP8.5, respectively. The increase in summer months are relatively small, mainly in the range of 9~12d and 8~10d for RCP4.5 and RCP8.5, respectively. Different from NHD18_5, HDD18_5 reflects decreases of energy demand in winter months and increase in summer months. Most winter months are included in both the present and future heating periods during which future warming has the dominant effect causing a decrease of HDD therefore a negative HDD18_5. For summer months, HDD5 is zero because it is beyond the present heating period, but HDD18 is not zero as there are days with temperature below 18 °C in the future. Therefore a positive summertime HDD18_5 would result from the policy change. Under RCP4.5, an increase in the range of 1~83 °C·d can be found during April-October; under RCP8.5, an increase would occur during May-August only, ranging from 22 to 56 °C·d. Specifically, the increase before June and after August in both NHD18_5 and HDD18_5 are mainly due to the increase over the heating areas except the Tibetan Plateau where temperature is cold and the heating period is long (therefore little change in both NHD18_5 and HDD18_5). During June, July and August, NHD5 (HDD5) is almost 0 over the Tibetan Plateau in the present day due to the defined heating period, but NHD18 (HDD18) accounts for the impact of several days with temperature below 18 °C. Therefore, at the end of the century under the high emission scenario, the maximum increase of NHD18_5 (HDD18_5) in summer is expected over the Tibetan Plateau (figures not shown for brevity).

### Changes weighted by population

**Figure 6:**
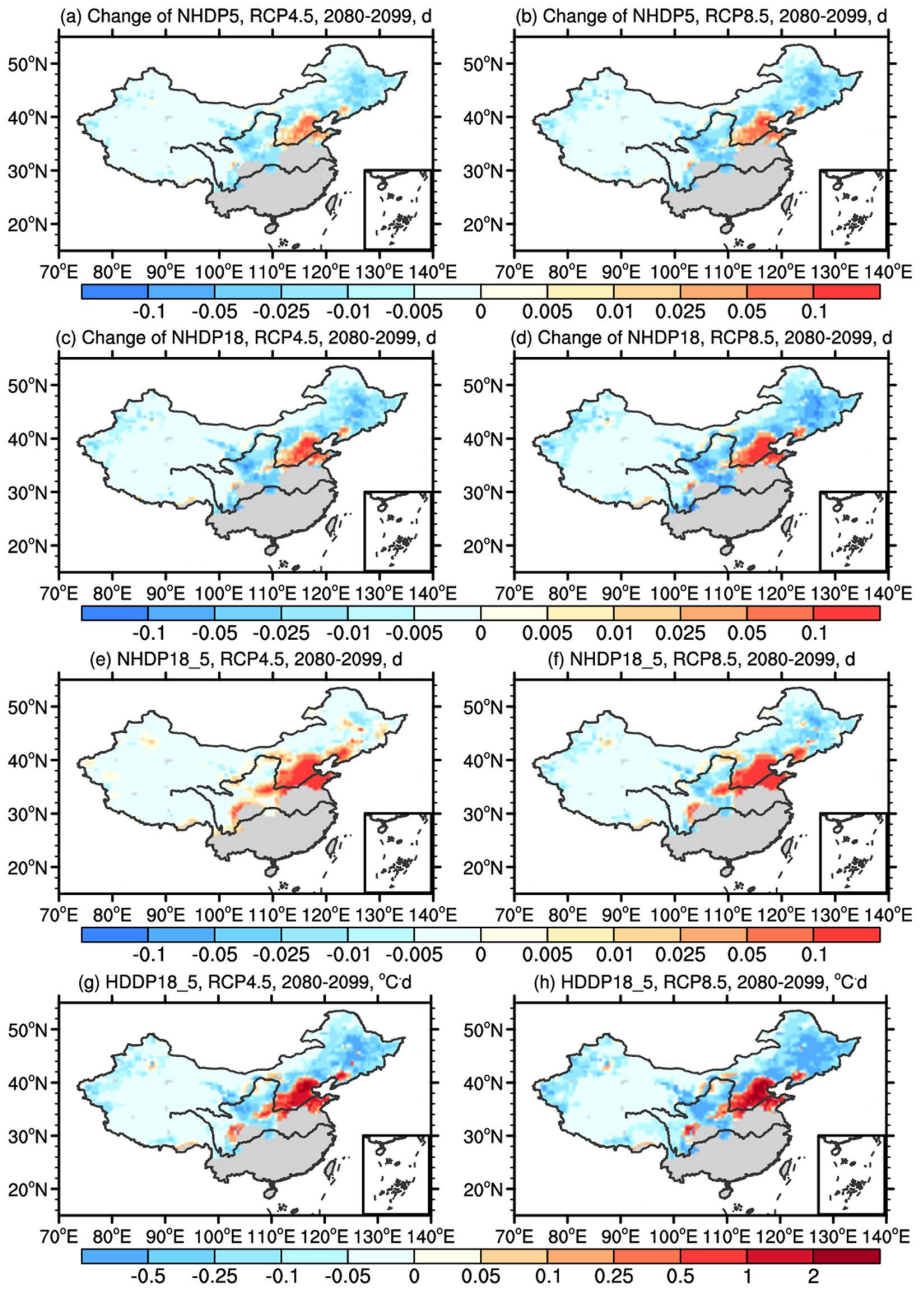
Changes of population weighted (**a**,**b**) NHD5, (**c**,**d**) NHD18, (**e**,**f**) NHD18_5, and (**g**,**h**) HDD18_5 (NHDP5, NHDP18, NHDP18_5 and HDDP18_5, respectively) in the end (2080–2099) of the 21st century under RCP4.5 and RCP8.5 compared to the present day (1986–2005) (unit: d&°C·d). The software NCL 6.2.0 (free software; http://www.ncl.ucar.edu/) was used to create the maps.

As heating demand is related to both HDD/NHD and population, here we also examine the changes weighted by the corresponding population in the same grid, hereafter referred to as NHDP5, NHDP18, NHDP18_5 and HDDP18_5. Spatial distributions of the changes under both scenarios are presented in Fig. 6. Large difference can be found when comparing Fig. 6 to Figs 3 and 5. In general, the changes of population weighted indices are more pronounced in the regions with higher population density (e.g., North China). For NHDP5 and NHDP18, the most noticeable difference compared to NHD5 and NHD18 is the increase in northern China despite the warming there. This increase is due to the large future population increase projected over the region, which overwhelms the effect of climate change. Other regions show slight decrease (within ± 0.01d) effected by the decrease of both the number of the heating days and population (Fig. 6a–d). As a result, the sum of NHDP5 and NHDP18 over the heating areas are −10d and −13d for RCP4.5, and are −17d and −22d for RCP8.5, respectively.

Similar results can also be found in NHDP18_5 and HDDP18_5, with the increased area extending to the surrounding regions. Compared to NHD18_5, decrease rather than increase in most parts can be found in NHDP18_5, which is caused by the decrease of population overwhelming the effect of potential policy change (Fig. 6e,f). Significant increase (greater than 2 °C·d) of HDDP18_5 is also shown in the regions with higher population density regardless whether HDD18_5 increases or decreases (Fig. 6g,h). The sum of NHDP18_5 and HDDP18_5 over the heating areas are 31d and −60 °C·d for RCP4.5, and 22d and −290 °C·d for RCP8.5, respectively. Note that the increase in both the heating degree days and the number of heating days in northern China indicates the heating energy consumption (in KW·h/m^2^) would increase regardless of whether the policy change will be implemented or not.

For the regional mean changes of the population-weighted indices, the changes in general follow that of the unweighted averages but with a smaller magnitude. For brevity the figures are not shown here.

### Decadal changes of boundary line for heating areas

**Figure 7:**
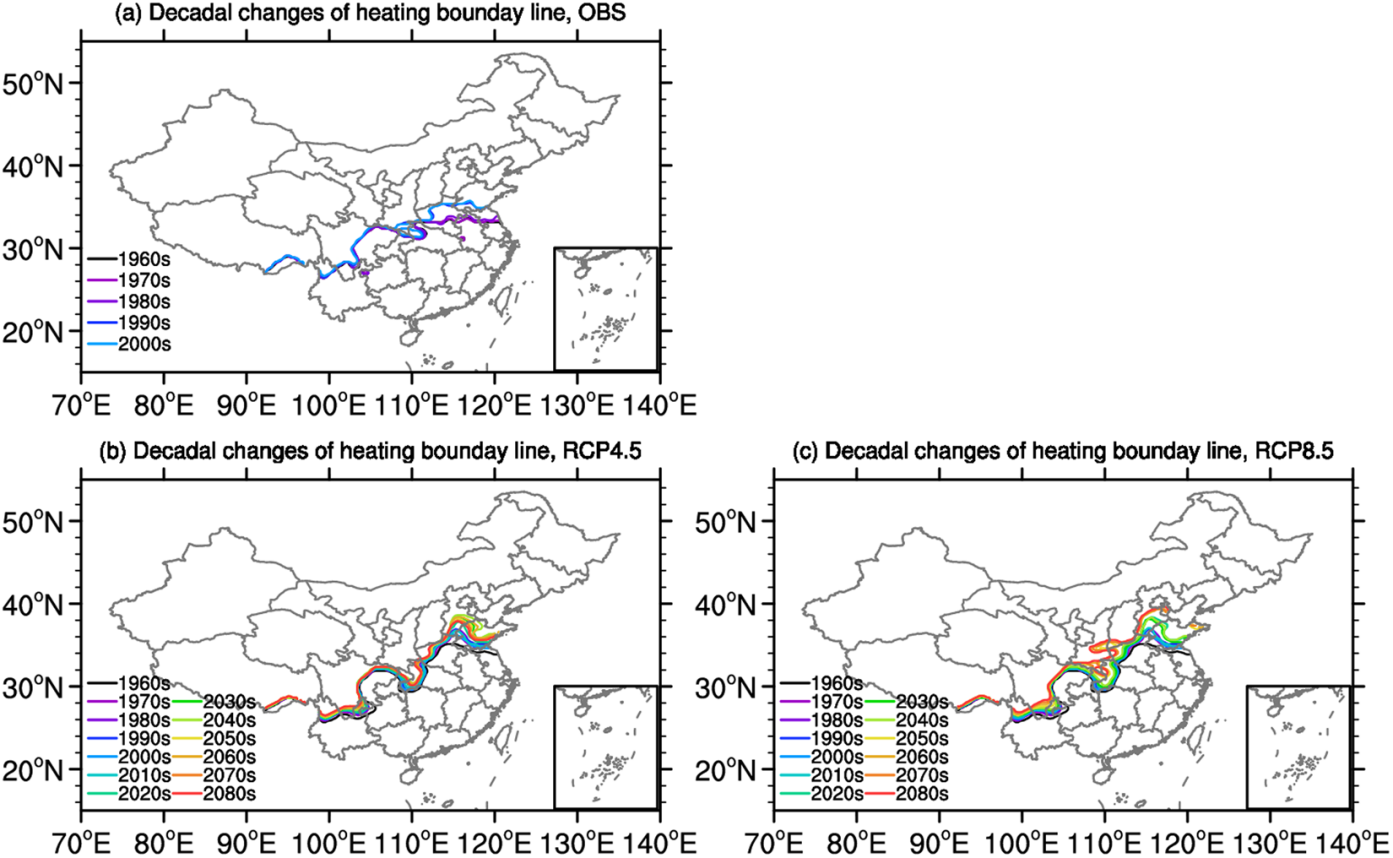
Decadal changes of observed and simulated heating boundary line ((**a)** observation; (**b**) RCP4.5; (**c**) RCP8.5). The software NCL 6.2.0 (free software; http://www.ncl.ucar.edu/) was used to create the map.

Following the current heating practice in China, decadal changes of the observed and simulated heating boundary line in present day and in the future under different scenarios are presented in Fig. 7. From the beginning of the 1960s, the observed heating line moves northward gradually. By 2000s, most parts of Henan, Anhui and southwestern part of Shandong Provinces have been excluded from the concentrated heating area. In addition, the heating line shows negligible changes in western part of China but migrated northward by about two degrees in the eastern part (Fig. 7a). Same as observations, the trend of simulated heating line in the present day climate also shows a northward shift (Fig. 7b). However, the simulated boundary is south of the observed in the west, and north of the observed in the east, as a result of the model’s cold and warm bias over the Tibetan Plateau and North China^29^. In the future, the heating line is projected to shift further north, faster under RCP8.5 than RCP4.5 (Fig. 7b,c). By the end of the century, under RCP4.5, southern Hebei will no longer meet the heating criteria, while under RCP8.5, southeastern Shan’xi, northern Shandong, southern Shanxi and Tianjin will be also excluded. In addition, changes in the western part are relatively small under the two scenarios due to the cold climate condition there.

**Figure 8:**
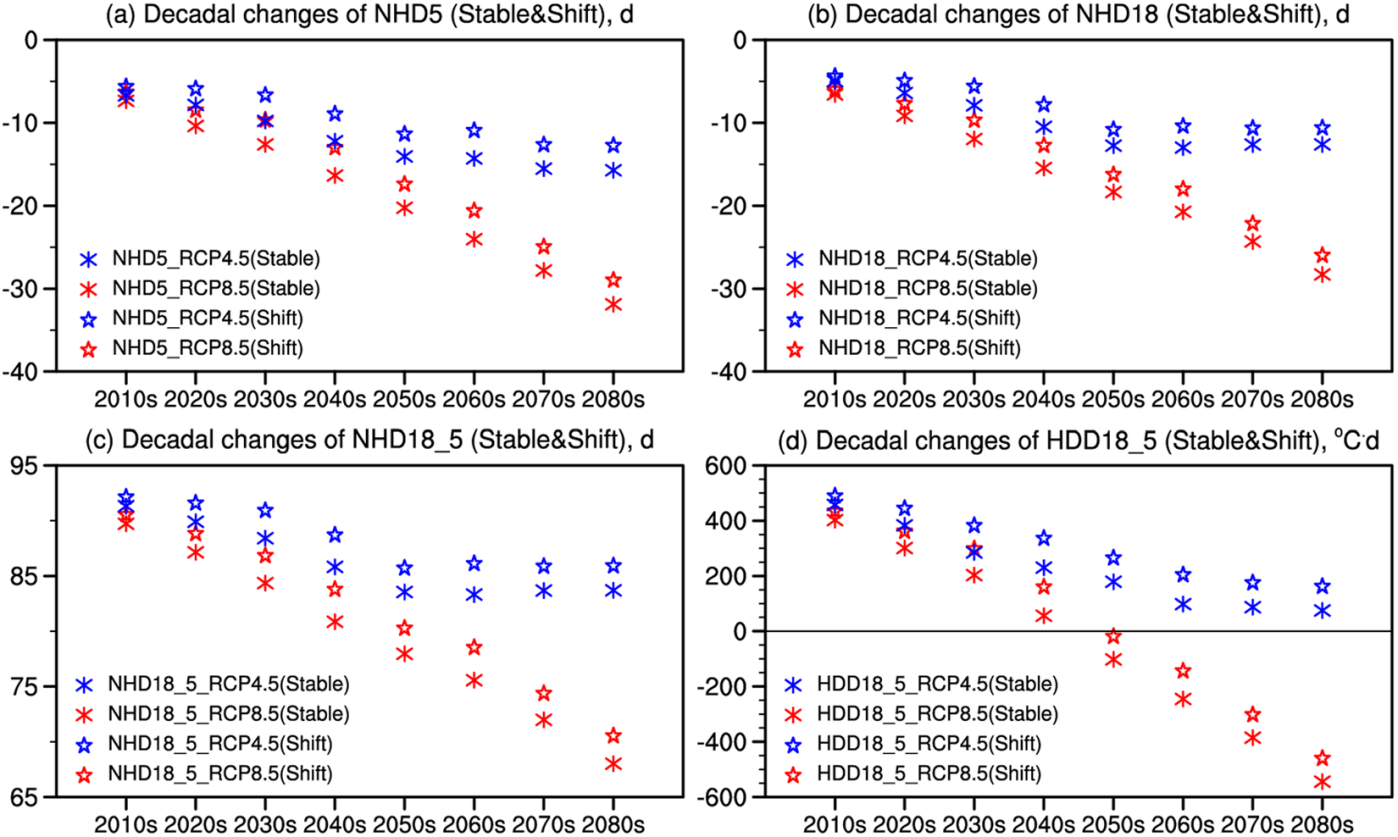
Decadal changes of (**a**) NHD5, (**b**) NHD18, (**c**) NHD18_5 and (**d**) HDD18_5 over the heating areas of China compared to the present day (1986–2005) (unit: d&°C·d). (Stable: not accounting for heating line shift; Shift: accounting for heating line shift).

Corresponding to the changes of heating line, decadal changes of regional mean number of the heating days in the heating areas with and without accounting for the heating line shift are presented in Fig. 8a,b. Whether the heating line shift is accounted for or not, the projected changes of NHD5 are similar; the magnitude of the projected decrease is slightly larger when the shift of heating line (therefore decrease of the heating area) is not accounted for. Differences in the changes of NHD5 with the heating line shift between RCP8.5 and RCP4.5 is less than 4d before 2050 s, while the difference can be up to a factor of ~2 (−29d vs. −13d) by 2080 s. The same performance can be found in the changes without the heating line shift, −6d vs. −7d in 2010s and −32d vs. −16d in 2080 s. Decrease of NHD18 from 2010 s to the end of the 21st century can also be found in both scenarios with greater decrease under RCP8.5. Specifically, changes before 2050 s differ little between the two scenarios; afterwards, especially by the end of the 21st century, they can differ by up to a factor of two, −11d vs. −26d (accounting for heating line shift) and −13d vs. −28d (not accounting for heating line shift) under RCP4.5 and RCP8.5, respectively.

Considering the probable future policy, decadal changes reflected by regional mean NHD18_5 and HDD18_5 over the heating areas of China are shown in Fig. 8c,d. Under RCP4.5, the heating degree days and the number of the heating days would increase whether the heating line shift is considered or not (shift or stable). Under RCP8.5, NHD18_5 is approximately 90 days in 2010 s (reflecting the immediate impact of policy shift), and decreases afterwards due to continuous warming but is still positive by the end of the century reflecting the dominant impact of policy shift on the heating period. However, the impact on heating energy demand is slightly different, especially under RCP8.5 in which the impact of warming dominating over the effects of policy shift by the second half of the century. Under RCP8.5, HDD18_5 is approximatey 400 °C·d in 2010 s (refleting the immediate impact of policy shift), decreases afterwards due to continuous warming, becomes zero by mid-century, and reaches approximately −600 °C·d by 2080 s. This is because greater warming under the high emission scenario RCP8.5 can lead to larger HDD18_5 decrease over cold regions like the Tibetan Plateau while little change can be found there in NHD18_5 as the temperature is well below 18 °C even with future warming.

## Conclusions and Discussions

Changes of the heating degree days and the number of the heating days under different thresholds and scenarios, as well as the heating boundary line are analyzed based on a high resolution regional simulation using a one-way nested regional climate model (RegCM4). The results are as follows:

1. RegCM4 can realistically reproduce the present-day number of the heating days under different thresholds. In general, a better model performance is found for NHD18 than for NHD5.
2. Significant decrease in both NHD5 and NHD18 are projected for the future in response to the greenhouse gases induced warming, with more pronounced changes under RCP8.5 than RCP4.5, in particular during the second half of the 21st century. Accounting for the impact of the potential policy change (as reflected by NHD18_5), NHD would increase under the two scenarios and the magnitude of the increase would taper off with time as warming increasingly compensates for the effect of policy changes. HDD18_5 show changes similar to what NHD18_5 reflects under RCP4.5; however, under RCP8.5, the impact of policy shift is dominant (as reflected by a positive HDD18_5 value) during the first half of the century only, and climate change effects dominate over policy change during the second half of the century. In addition, changes reflected by the regional mean NHD18_5 indicate an increase of heating days in spring, summer and autumn and little change in winter while HDD18_5 shows an increase of heating energy demand in summer and a decrease in winter.
3. Significant increase in population-weighted NHDP5 and NHDP18 can be found in parts of northern China due to the increased population there, which overwhelms the effect of climate change. Similar results can also be found in NHDP18_5 and HDDP18_5, with the increased area extending to the surrounding regions. This illustrates the importance of considering the population distribution to the estimates of climate change impacts on energy demand.
4. The model can reproduce well the heating boundary line, with a slight northward and southward spatial bias over the eastern and western parts, respectively, due to temperature biases in those areas. Following the time evolution, a northward migration is projected with a greater change of ~2 degree under RCP8.5 than RCP4.5.

With the rapid socio-economic development, the current criteria in central heating areas might be changed in the future. However, based on our analysis, change of the threshold for heating degree days plays a more important role in summer months compared to winter months. Heating demand in winter is unambiguous, and shows no sensitivity to how the heating degree days are calculated. On the other hand, fewer days with the temperature below 18 °C are anticipated under global warming. Therefore, in terms of emission reduction policies, whether or not to take the policies adopted in developed countries should be considered seriously.

Finally, as a discussion point, energy consumption changes due to temperature change might be small compared to those due to other factors, such as changes in income and technology^19^. In addition, the issue of future energy demand due to changes in heating degree days and the number of the heating days is complex and multifaceted and in this paper, we only adopted simple assumptions for the relation between the indices, population distribution and energy demand and did not consider the increase of cooling degree days which might offset the decrease of heating demand under future warming. Moreover, although the high-resolution regional climate model shows remarkable improvements in reproducing the present day climate compared to the driving global climate model, large uncertainties still exist in the projection of future climate changes, especially at the regional and local scales. Based on output from one RCM alone, this study is subject to uncertainties related to potential model dependency. To address uncertainties in climate projection, further research making use of multiple models and the indices including both HDD and CDD is necessary to support a more comprehensive assessment of energy demand within a multi-model framework (e.g. CORDEX)^32^.

## Methods

### Data

The observational dataset of CN05.1^31^ used in this study is an augmentation of CN05^33^ and more stations data (2416 vs. 751) are included. Both CN05 and CN05.1 were generated using the same interpolation approach as the Climatic Research Unit dataset (CRU)^34^. Three resolutions of 0.25°, 0.5° and 1° are available in CN05.1, and we use the 0.5° resolution to validate the model in this study.

The model simulations are conducted using RegCM4 driven by boundary conditions from the BCC_CSM1.1. The simulation domain includes the whole China continent and surrounding areas with a grid spacing of 50 km. The period of the simulations is from 1951 to 2099, with observed GHG concentrations in 1951–2005 and under the RCP4.5 and RCP8.5 scenarios in 2006–2099.

The population data is developed by the International Institude for Applied System Analysis (IIASA) (GGI Scenario Database v.2.0, available at www.iiasa.ac.at/Research/GGI/DB)^35^. The resolution of the dataset is 0.5° × 0.5° (longitude-latitude). Three scenarios of population growth are provided in the dataset, that is, A2, B2 and B1. By comparing the dataset with that under RCP scenarios at the country level^36^, we selected A2 for RCP8.5 scenario and B1 for the RCP4.5. Future changes of total population in China shows a continuous growth under A2 and growth followed by a decline in the B1. But for the spatial distribution, changes under the two scenarios are similar, characterized by an increase in North China, the Yangtze River Basin and southern coastal areas as well as a decrease (or little change) elsewhere.

### Heating degree days and the number of the heating days

HDD is a metric reflecting the demand for energy to heat a building. It is defined as the difference between the daily mean temperature and a base temperature (which is considered as a temperature for human comfort) accumulated over a certain period. In China, according to the code for Design of Heating Ventilation and Air Conditioning (GB50019-2003)^23^, the reference temperature for HDD is 18 °C, which is widely used in the world. However, the policy for room heating is more stringent. Specifically, only regions meeting several conditions (as elaborated below) can be heated to the reference temperature and the starting and ending dates of the heating period (DSH and DEH) are also different among provinces, leading to tens of DSH and DEH in the country.

The conditions for the current heating in China can be summarized as follows: 1) only areas with more than 90 days of below 5 °C temperature in an annual cycle from September 1 to August 31 can be heated; 2) the heating starts (ends) when temperature is below (above) 5 °C for a continuous 5d period. According to the criteria, four indices are used and defined as:

- *HDD5*: Sum of absolute difference between daily mean temperature (TM) and 18 °C where TM below 18 °C (if DEH-DSH > 90).
- *HDD18*: Sum of absolute difference between TM and 18 °C where TM < 18 °C.

- *NHD5*: Number of days where TM below 18 °C (if DEH-DSH > 90).
- *NHD18*: Number of days where TM below 18 °C.

HDD5 and NHD5 define the heating degree days and the number of the heating days reflecting the current practice in China. HDD18 and NHD18 define the heating degree days and the number of the heating days according to common practices in the developed world, i.e., whenever temperature drops below 18 °C, the heating degree days or the number of the heating days will be accounted for.

It should be noted that in the calculation, the annual cycle is from September 1 to August 31 instead of the calendar year to encompass a continuous heating season. In addition, while HDD18 and NHD18 can be estimated for any area, in this study it is calculated only within the centralized heated areas in China (i.e., areas with temperature below 5 °C for more than 90 days a year) to be consistent with the current state of heating infrastructure.

### Boundary line for heating areas

Historically, the Qinling-Huaihe line has generally been accepted as the boundary for heating, although some (albeit limited) areas north of the line are not on central heating. In our analysis, the heating areas are defined based on the criteria described in the last Section, and therefore the boundary line slowly changes as the global warming progresses. However, in calculating the number of the heating days in the future we neglect the impact of boundary line changes, because these changes are small and slow as evident from the decadal changes of the heating boundary line in “Results” Section. Considering the effects of climate natural variability, the decadal changes are based on 20-year periods that are ten years apart with a 10 year overlapping period, e.g. 1960 s = 1961–1980, 1970 s = 1971–1990.
